# Foot-and-mouth disease virus non-structural protein 3D induces necroptosis through interaction with RIPK3 in PK-15 and BHK-21 cells

**DOI:** 10.3389/fvets.2026.1872229

**Published:** 2026-07-23

**Authors:** Zhixiong Zhang, Juanbin Yin, Mengting Zhang, Xiaoxiao He, Wenjing Zhao, Guo Ji, Shijun Bao

**Affiliations:** 1 College of Veterinary Medicine, Gansu Agricultural University, Lanzhou, China; 2 State Key Laboratory of Animal Disease Control and Prevention, Lanzhou Veterinary Research Institute, Chinese Academy of Agricultural Sciences, Lanzhou, China

**Keywords:** FMDV, 3D, necroptosis, RIPK3, MLKL

## Abstract

**Introduction:**

Foot-and-mouth disease (FMD) is a highly contagious infectious disease caused by foot-and-mouth disease virus (FMDV). FMDV replicates not only in epithelial cells, where it induces vesicular lesions, but also in myocardial and skeletal muscle tissues, leading to myocarditis and even fatal outcomes in infected animals. RIPK3/MLKL-mediated necroptosis is a proinflammatory cell death process that plays a significant role in viral infections. However, whether FMDV infection can trigger necroptosis in host cells remains unclear.

**Methods:**

We assessed necroptosis induced by FMDV infection through activation of the RIPK3/MLKL pathway in PK-15 and BHK-21 cells. Specific inhibitors GSK’872 (targeting RIPK3 phosphorylation) and NSA (targeting MLKL phosphorylation) were applied to examine the impact of this pathway on viral replication. Additionally, Co-immunoprecipitation (Co-IP) was used to analyze the interactions between FMDV-encoded proteins and RIPK3/MLKL, and AlphaFold3 was used to predict the interaction interface between the 3D protein and the RHIM-containing region of RIPK3.

**Results:**

FMDV infection activated the RIPK3/MLKL pathway and induced necroptosis in both PK-15 and BHK-21 cells. Inhibitor assays revealed that blocking RIPK3 or MLKL phosphorylation significantly reduced FMDV VP1 expression and viral titers, indicating that the necroptosis pathway favors FMDV replication. Further investigation revealed that the FMDV non-structural protein 3D is the key protein responsible for activating RIPK3/MLKL pathway. Moreover, we confirmed that the 3D protein interacts and colocalizes with RIPK3. AlphaFold3 structural prediction suggested that 3D can directly associate with the RHIM-containing region of RIPK3, with residues R320, H322, E324, and Y346 of 3D interacting with residues N466, V469, and K471 of RIPK3 at the interface.

**Discussion:**

Our results demonstrate that FMDV infection induces necroptosis in host cells. This finding expands the modes of host cell death caused by FMDV infection, advances the understanding of the role of necroptosis in viral infections, and may help elucidate the pathogenic mechanisms of FMDV.

## 1 Introduction

Foot-and-mouth disease (FMD) represents a significant global animal health issue. This transboundary livestock disease affects both wild and domesticated artiodactyls (cloven-hoofed animals) such as pigs, cows, and sheep (1). FMD poses a considerable threat to local and national economies, often leading to reduced animal productivity, increased production costs, and limited access to markets (2). The disease is caused by the foot-and-mouth disease virus (FMDV), which belongs to the genus *Aphthovirus* within the family *Picornaviridae* and possesses a single-stranded positive-sense RNA genome. There are seven distinct serotypes of FMDV: SAT1, SAT2, SAT3, Asia 1, O, A, and C (3). The FMDV genome comprises a long 5’untranslated region (5’UTR), an open reading frame (ORF), and a short 3’UTR, forming an 8.3 kb single-stranded positive-sense RNA. The virus employs host translation mechanisms to produce a polyprotein, which is subsequently cleaved into four structural proteins (VP1, VP2, VP3, VP4) and eight non-structural proteins (L, 2A, 2B, 2C, 3A, 3B, and 3D) (4). The non-structural protein 3D, an RNA-dependent RNA polymerase (RdRp), synthesizes negative-sense RNA using the viral genomic RNA as a template within the replication complex (RC). This negative-sense RNA is then used to produce more positive-sense RNA (4, 5). However, a growing number of studies have demonstrated its non-polymerase roles, including modulation of host cell biological processes, viral replication complex assembly and localization, autophagy, and innate immune responses (6).

Necroptosis is a form of regulated cell death that modulates pathogen-mediated host immune defenses by inducing inflammatory responses. This intricate balance between induction of inflammatory responses and clearance of pathogens via ‘suicidal death’ is a noteworthy contribution to host–pathogen standoff, as it determines the degree of pathogenic invasion and pathogenicity (7). Necroptosis signalling is mediated through the formation of a multiprotein complex-the so-called necrosome-in which receptor interacting protein serine/threonine kinase 3 (RIPK3) plays a major effector role. In the absence of caspase-8-mediated apoptosis, tumour necrosis factor (TNF) induces the assembly of the canonical necrosome complex composed of two RIPKs: RIPK1 and the effector RIPK3 (8–10). More recently, necroptosis has been recognized as an antiviral mechanism after virus infections, such as murine cytomegalovirus (MCMV) (11), herpes simplex virus-1 (HSV-1) (12, 13), vaccinia virus (VACV) (14) and influenza A virus (IAV) (15), by forming a non-canonical necrosome composed of RIPK3 and an interferon-stimulated gene (ISG), Z-DNA-binding protein 1 (ZBP1) (16, 17). RIPK3-mediated phosphorylation of its substrate, mixed lineage kinase domain-like pseudokinase (MLKL), triggers executional signals that increase membrane permeabilization for the release of intracellular damage-associated molecular patterns and thereby elicits a robust antiviral immune response (18–20).

RIPK3 autophosphorylation is the primary prerequisite for its self-activation to relay downstream signals for inducing necroptotic cell death; however, this fundamental mechanism remains hypothetical. It has been proposed that intermolecular interactions mediated by the RIP homotypic interaction motif (RHIM) result in high-order hetero-amyloid structures, which may serve as a platform for RIPK3 autophosphorylation (21–23). Recent findings have also revealed that artificially induced RIPK3 homo-oligomerization is sufficient to induce necroptosis (24, 25), which suggests that the kinase activity of RIPK3 could be activated by proximity within RIPK3 oligomers. In addition, RIPK3 assembles into discrete functional amyloid-like foci in the cytosol; however, it remains unclear how RIPK3 amyloid formation contributes to necroptosis signaling (7, 26, 27).

In this study, we investigated whether FMDV infection can induce necroptosis in host cells. We demonstrated that FMDV infection activates the RIPK3/MLKL pathway to trigger necroptosis in both PK-15 and BHK-21 cells, and further identified that the FMDV non-structural protein 3D is the key protein responsible for activating this pathway. In addition, we identified that the 3D protein interacts and colocalizes with RIPK3. Our findings not only expand the modes of host cell death caused by FMDV infection, but also further enrich our understanding of the role of necroptosis in viral infections.

## 2 Materials and methods

### 2.1 Cell lines and viruses

PK-15 (pig kidney cells, ATCC CCL-33), BHK-21 (baby hamster kidney fibroblasts, ATCC CCL-10) and HEK-293T (human embryonic kidney 293T, ATCC CRL-11268) cells were obtained from Lanzhou veterinary research institute. All cell lines were cultured in Dulbecco’s modified Eagle’s medium (DMEM, Jianshun, China). All mediums were supplemented with 10% fetal bovine serum (FBS, Gibco, United States), 1% streptomycin (0.2 mg/mL) and penicillin (200 U/mL). All cells were maintained at 37 °C with 5% CO₂.

The FMDV serotype O strain was obtained from the National Foot-and-Mouth Disease Reference Laboratory, Lanzhou Veterinary Research Institute, Chinese Academy of Agricultural Sciences. All experiments involving FMDV were conducted in a Biosafety Level 3 (BSL-3) laboratory.

### 2.2 Plasmids and antibodies

The VP0, VP1, VP2, VP3, 2B, 2C, 3A, 3C, 3D, and L genes were cloned into the pCMV-Flag vector to obtain VP0-Flag, VP1-Flag, VP2-Flag, VP3-Flag, 2B-Flag, 2C-Flag, 3A-Flag, 3C-Flag, 3D-Flag, and L-Flag, respectively. The RIPK3 and MLKL genes were cloned into the pcDNA3.1-Myc vector to obtain RIPK3-Myc and MLKL-Myc. All constructed plasmids were analyzed and verified by DNA sequencing. Mouse anti-Flag (Sigma-Aldrich, Cat #F1804, 1:5000), Rabbit anti-Myc (Sigma-Aldrich, Cat #M5546, 1:5000), Mouse anti-β-actin (Sigma-Aldrich, Cat #A5441, 1:5000), Rabbit anti-GAPDH (Cell Signaling Technology, Cat #2118S, 1:5000), Rabbit anti-RIPK3 (Cell Signaling Technology, Cat #10188T, 1:1000), Rabbit anti-MLKL (Proteintech, Cat #21066-1-AP, 1:1000), Rabbit anti-phosphorylation RIPK3 (Abcam, Cat #ab222320, 1:1000), Rabbit anti-phosphorylation MLKL (Abcam, Cat #ab196436, 1:1000), Goat anti-mouse IgG/Alexa Fluor 594 (Invitrogen, Cat # A11005, 1:500), Goat anti-rabbit IgG/Alexa Fluor 488 (Invitrogen, Cat # A11008, 1:500), Goat anti-rabbit IgG/Alexa Fluor 594 (Invitrogen, Cat # A11037, 1:500), and Goat anti-mouse IgG/Alexa Fluor 488 (Invitrogen, Cat # A28175, 1:500) were purchased from the indicated manufacturers. Mouse anti-VP1 antibody (1:1000) was obtained from the Lanzhou Veterinary Research Institute.

### 2.3 Viral infection

PK-15 or BHK-21 cells in good growth condition were trypsinized into a single-cell suspension and seeded into cell culture dishes or plates. When the cells reached 80–90% confluence, the old medium was removed, and the cells were washed with PBS, followed by the addition of maintenance medium containing 2% FBS. The cells were then infected with FMDV, and a blank control group was set up simultaneously. At different time points post-infection, the cells and/or supernatants were collected to prepare protein or RNA samples for subsequent assays.

### 2.4 Virus titers assay

Viral infectivity was quantified by the endpoint dilution assay. The virus was serially diluted and used to infect BHK-21 cells in a 96-well plate, and the 50% tissue culture infectious dose (TCID₅₀) was then calculated using the Reed–Muench method.

### 2.5 Western blot

The collected protein samples were lysed and denatured, then subjected to SDS-PAGE. After electrophoresis, the proteins were transferred onto a PVDF membrane. The membrane was blocked with 5% non-fat milk for 2 h at room temperature, followed by overnight incubation with the corresponding primary antibodies at 4 °C. The membrane was then washed three times with TBST and incubated with the corresponding secondary antibodies for 1 h at room temperature. After final washing, the signals were visualized using chemiluminescent substrate. Gray values of target protein bands were normalized to the corresponding loading control (β-actin for PK-15 cells; GAPDH for BHK-21 cells) using ImageJ software. The normalized ratio for each sample was then expressed relative to the 0 h time point control.

### 2.6 RNA extraction and qPCR

Total cellular RNA was extracted using the QIAGEN RNA extraction kit. Briefly, the culture medium was removed from the dishes, and 600 μL of RLT buffer (containing 1% β-mercaptoethanol) was added to lyse the cells. An equal volume of 70% ethanol was then added, and the mixture was vortexed. The liquid was transferred to an RNeasy Mini spin column and centrifuged. After washing and elution, the total RNA samples were obtained. The RNA samples were reverse transcribed using the PrimeScript™ RT reagent Kit with gDNA Eraser (Perfect Real Time) (RR047A, Takara). Finally, real-time quantitative PCR analysis was performed using the QuantiNova™ SYBR Green PCR Kit (QIAGEN). The qPCR primers were as follows: Porcine RIPK3 forward primer (F): 5′-GCCATCTTCACTCCCAAGGA-3′; Porcine RIPK3 reverse primer (R): 5′-TGTTGCTGAACTGG TCCCTC-3′. Porcine MLKL forward primer (F): 5′-ACGGCTACAGAGACGAAGTG-3′; Porcine MLKL reverse primer (R): 5′-TGCCGTTGTAGTTGATGCCC-3′. Porcine β-actin forward primer (F): 5′-TGCGGGACATCAAGGAGAAG-3′; Porcine β-actin reverse primer (R):5′ TGCCGTTGTAGT TGATGCCC-3′. Relative mRNA expression levels were normalized to the expression of β-actin and relative mRNA expression levels were calculated using the threshold cycle (2^−ΔΔCt^) method.

### 2.7 Indirect immunofluorescence assay

After the pretreated cells reached the indicated time points, the culture medium was removed, and the cells were washed once or twice with PBS. The cells were then fixed with 4% paraformaldehyde for 15 min at room temperature, followed by two washes with PBS. Subsequently, the cells were permeabilized with PBS containing 0.5% Triton X-100 for 10 min at room temperature and blocked with 5% bovine serum albumin 30 min. The cells were then incubated with primary antibodies overnight at 4 °C. After three washes, the cells were incubated with fluorescent secondary antibodies for 1 h at room temperature in the dark, followed by DAPI staining for 10–15 min to label the nuclei. Finally, after washing, images were acquired using a Leica confocal microscope.

### 2.8 Co-immunoprecipitation

HEK-293T cells were seeded in 60 mm dishes. When the cells reached 80–90% confluence, they were transfected with RIPK3-Myc/MLKL-Myc, 3D-Flag alone, or co-transfected with RIPK3-Myc/MLKL-Myc and 3D-Flag. At 24 h post-transfection, protein samples were collected and lysed in 600 μL of IP lysis buffer at 4 °C for 40 min. One-tenth of the lysate was taken as input to verify protein expression. The remaining lysate was incubated with the appropriate antibody for immunoprecipitation overnight at 4 °C, followed by incubation with pre-equilibrated protein A/G beads for approximately 4 h. Finally, the samples were eluted with 2 × protein loading buffer and analyzed by Western blot.

### 2.9 Statistical analysis

Student’s t-tests statistical analysis was performed using GraphPad Prism 8 software (GraphPad), and the difference was statistically significant at *p* < 0.05 (*, *p* < 0.05; **, *p* < 0.01; ***, *p* < 0.001; ****, *p* < 0.0001; ns, not significant). Data are presented as mean ± standard deviation (SD). Each experiment was independently repeated at least three times.

## 3 Results

### 3.1 FMDV infection induces necrosis in PK-15 cells

Cell death is essential for various physiological processes in multicellular organisms, and manipulating programmed cell death is one of the major mechanisms by which pathogens survive within the host. Many viral infections cause cytopathic effects in host cells, leading to cell membrane rupture, which facilitates viral replication and spread. It is known that CF 488A-labeled Annexin V binds to phosphatidylserine (PS) exposed on the cell surface, staining apoptotic cells green. Ethidium homodimer III (EthD-III) is a highly positively charged nucleic acid probe that is impermeable to live cells but stains cells with compromised membrane integrity (necrotic cells) red. FMDV infection of PK-15 or BHK-21 cells induced obvious cytopathic effects. To further determine whether FMDV infection causes necrosis in host cells, we infected PK-15 cells with FMDV (MOI = 1). At 6 h post-infection, cells were stained with CF 488A and EthD-III. Fluorescence microscopy revealed that PK-15 cells infected with FMDV were stained red by EthD-III, whereas no obvious green fluorescence was observed with CF 488A staining (Figure 1). These results indicate that FMDV infection induces necrosis in PK-15 cells.

**Figure 1:**
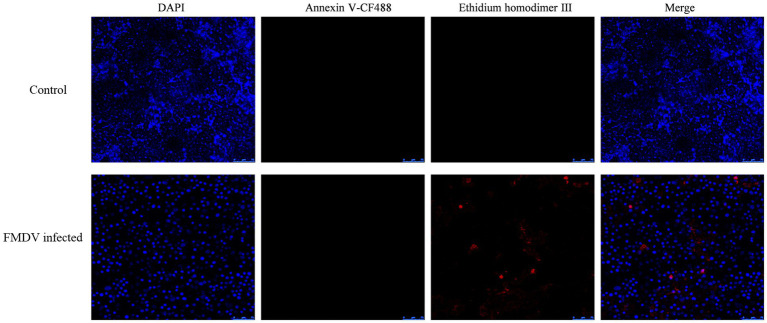
FMDV infection induces necrosis in PK-15 cells. Uninfected and FMDV-infected (6 h) PK-15 cells were stained with Annexin V-CF488 for apoptotic cells (green) and EthD-III for necrotic cells with compromised membrane integrity (red), and nuclei were counterstained with DAPI (blue). Stained cells were then examined under a fluorescence microscope.

### 3.2 FMDV infection activates the necroptosis pathway in PK-15 cells

FMDV infection can induce multiple modes of cell death. To further investigate the relationship between FMDV-induced necrosis and necroptosis, we infected PK-15 cells with FMDV (MOI = 1) and collected protein samples at 0, 3, 6, and 9 hpi. Western blot analysis revealed that the phosphorylated protein levels of RIPK3 and MLKL increased in a time-dependent manner (Figures 2A–C), whereas the total protein levels of RIPK3 and MLKL decreased at later time points post-infection (Figure 2A). In addition, total RNA was extracted at 3, 6, and 9 hpi, and the mRNA levels of RIPK3 and MLKL were measured by RT-qPCR. Compared with the control group, the RIPK3 mRNA level showed no significant change at 3 and 6 hpi, but decreased significantly at 9 hpi (Figure 2D). In contrast, the MLKL mRNA level was significantly upregulated at 6 hpi, which was not synchronized with the transcriptional changes of RIPK3 (Figure 2E). Indirect immunofluorescence analysis revealed that FMDV infection led to the formation of punctate aggregates of P-MLKL on the cell surface (Figure 2F). Collectively, these results demonstrate that FMDV infection activates the RIPK3/MLKL pathway, thereby inducing necroptosis in PK-15 cells.

**Figure 2:**
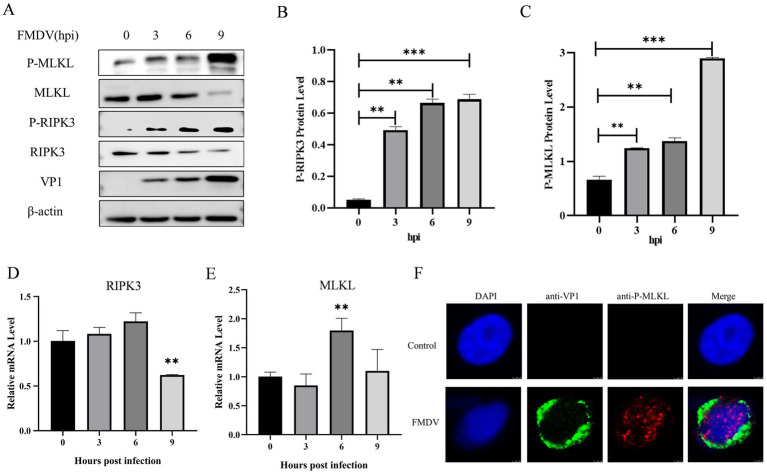
FMDV infection activates the necroptosis pathway in PK-15 cells. **(A)** Western blot analysis of P-MLKL, MLKL, P-RIPK3, RIPK3, VP1, and β-actin protein levels in PK-15 cells at 0, 3, 6, and 9 h post-FMDV infection. **(B)** The analysis of the gray value ratio of P-RIPK3/β-actin (normalized to 0 h control) in PK-15 cells. **(C)** The analysis of the gray value ratio of P MLKL/β-actin (normalized to 0 h control) in PK-15 cells **(D)** The RT-qPCR analysis of RIPK3 mRNA levels in PK-15 cells at 0, 3, 6, and 9 h post-FMDV infection. **(E)** The RT-qPCR analysis of MLKL mRNA levels in PK-15 cells at 0, 3, 6, and 9 h post-FMDV infection. **(F)** Indirect immunofluorescence detection of P-MLKL expression on the cell surface in FMDV-infected PK-15 cells compared to normal cells. Data were presented as the mean ± standard deviation from three independent experiments. Statistical significance was denoted as follows: **, *p* < 0.01; and ***, *p* < 0.001.

### 3.3 FMDV infection activates the necroptosis pathway in BHK-21 cells

To determine whether FMDV-induced necroptosis is specific to PK-15 cells, we further employed BHK-21 cells as an additional infection model. we infected BHK-21 cells with FMDV (MOI = 1), and collected protein samples at 0, 2, 4, and 6 hpi. The protein expression levels of P-RIPK3 and P-MLKL were detected by Western blot. The results showed that the expression levels of P-RIPK3 and P-MLKL increased in a time-dependent manner (Figures 3A–C), whereas the total protein levels of RIPK3 and MLKL decreased at later time points post-infection (Figure 3A). Furthermore, indirect immunofluorescence revealed that FMDV infection also induced punctate aggregation of P-MLKL on the surface of BHK-21 cells (Figure 3D). Collectively, these results indicate that FMDV infection similarly activates the RIPK3/MLKL pathway to induce necroptosis in BHK-21 cells.

**Figure 3:**
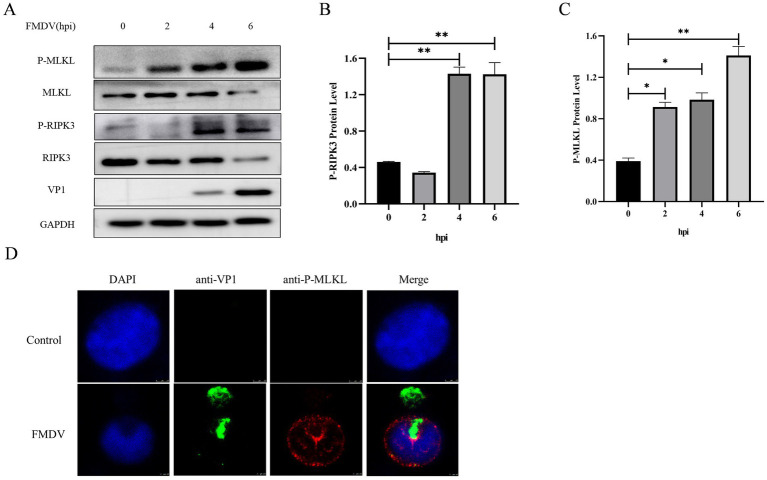
FMDV infection activates the necroptosis pathway in BHK-21 cells. **(A)** Western blot analysis of P-MLKL, MLKL, P-RIPK3, RIPK3, VP1, and GAPDH protein levels in BHK-21 cells at 0, 2, 4, and 6 h post-FMDV infection. **(B)** The analysis of the gray value ratio of P-RIPK3/GAPDH (normalized to 0 h control) in BHK-21 cells **(C)** The analysis of the gray value ratio of P-MLKL/GAPDH (normalized to 0 h control) in BHK-21 cells. **(D)** Indirect immunofluorescence detection of P-MLKL expression on the cell surface in FMDV-infected BHK-21 cells compared to normal cells. Data were presented as the mean ± standard deviation from three independent experiments. Statistical significance was denoted as follows: *, *p* < 0.05; and **, *p* < 0.01.

### 3.4 Inhibition of RIPK3 or MLKL phosphorylation in the necroptosis pathway suppresses FMDV replication

To investigate whether inhibition of the RIPK3/MLKL pathway affects FMDV replication in PK-15 and BHK-21 cells, PK-15 or BHK-21 cells were treated with GSK’872 (a specific inhibitor of RIPK3 phosphorylation) or NSA (a specific inhibitor of MLKL phosphorylation), respectively, followed by FMDV infection. In PK-15 cells, compared with the FMDV-infected control group, the GSK’872 + FMDV group exhibited significantly decreased RIPK3 phosphorylation and markedly reduced FMDV VP1 protein expression; the NSA + FMDV group showed significantly decreased MLKL phosphorylation and markedly reduced VP1 expression (Figure 4A). In addition, FMDV titers were significantly reduced in both the GSK’872 + FMDV and NSA + FMDV groups, whereas no significant change was observed in the DMSO + FMDV group (Figure 4B). In BHK-21 cells, similar results were obtained: GSK’872 treatment significantly decreased RIPK3 phosphorylation and VP1 expression, and NSA treatment significantly decreased MLKL phosphorylation and VP1 expression compared with the FMDV-infected control (Figure 4C). Consistently, viral titers were significantly lower in the GSK’872 + FMDV and NSA + FMDV groups, while the DMSO + FMDV group remained unchanged (Figure 4D). Collectively, these results demonstrate that inhibition of RIPK3 phosphorylation or MLKL phosphorylation in the necroptosis pathway suppresses FMDV replication in both PK-15 and BHK-21 cells.

**Figure 4:**
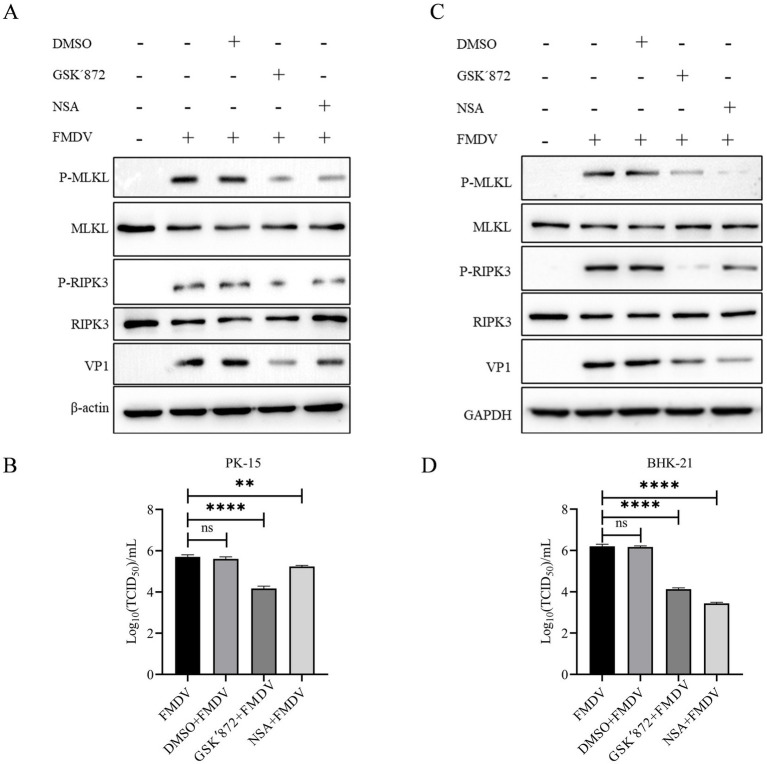
Inhibition of RIPK3 or MLKL phosphorylation suppresses FMDV replication in PK-15 and BHK-21 cells. **(A)** Western blot analysis of FMDV VP1, RIPK3, phosphorylated RIPK3 (P-RIPK3), MLKL, phosphorylated MLKL (P-MLKL), and β-actin in PK-15 cells. The lanes represent the mock control, FMDV-infected alone, and FMDV-infected cells treated with DMSO, GSK’872 (10 μM), or NSA (10 μM), respectively. **(B)** Viral titers in PK-15 cells. Cells were infected with FMDV and left untreated or treated with DMSO, GSK’872 (10 μM), or NSA (10 μM), and samples were collected at 6 h post-infection for titer determination. **(C)** Western blot analysis of FMDV VP1, RIPK3, P-RIPK3, MLKL, P-MLKL, and GAPDH in BHK-21 cells. The groups are the same as described in **(A)**. **(D)** Viral titers in BHK-21 cells. Cells were infected with FMDV and treated as in **(B)**, and samples were collected at 6 h post-infection for titer determination. Data were presented as the mean ± standard deviation from three independent experiments. Statistical significance was denoted as follows: ns, *p* > 0.05; **, *p* < 0.01; and ****, *p* < 0.0001.

### 3.5 FMDV 3D protein activates the RIPK3/MLKL signaling pathway

Previous results indicated that FMDV infection activates the RIPK3/MLKL signaling pathway. To investigate the effect of FMDV-encoded viral proteins on the RIPK3/MLKL pathway, PK-15 cells were transfected with the structural proteins VP0, VP1, VP2, VP3, as well as the non-structural proteins 2B, 2C, 3A, 3C, 3D, and L, respectively. Western blot analysis revealed that the non-structural protein 3D upregulated the phosphorylation level of MLKL (Figure 5A). To further validate this finding, empty vector or 3D-FLAG was overexpressed, and the results showed that 3D significantly promoted the upregulation of both RIPK3 and MLKL phosphorylation (Figures 5B–D). Collectively, these results demonstrate that the FMDV 3D protein activates the RIPK3/MLKL signaling pathway.

**Figure 5:**
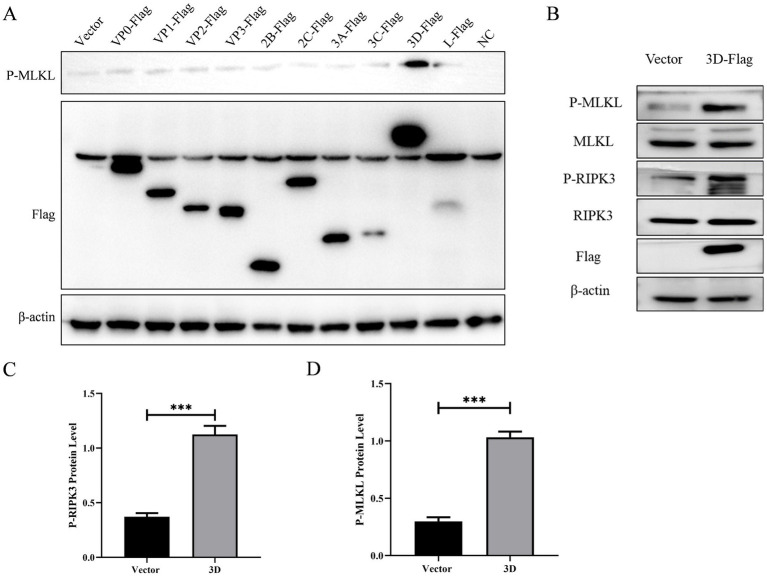
FMDV 3D protein activates the RIPK3/MLKL signaling pathway. **(A)** PK-15 cells were seeded in 6-well plates and cultured for 12 h, then transfected with 1 μg of plasmids encoding VP0-Flag, VP1-Flag, VP2-Flag, VP3-Flag, 2B-Flag, 2C-Flag, 3A-Flag, 3C-Flag, 3D-Flag, and L-Flag, or with empty vector as a control. At 24 h post-transfection, protein lysates were collected, and the expression of the Flag-tagged proteins and P-MLKL was analyzed by Western blotting. **(B)** PK-15 cells were seeded in 35-mm dishes and cultured for 12 h, then transfected with 1 μg of plasmid encoding 3D-Flag or with empty vector as a control. At 24 h post-transfection, protein lysates were prepared and analyzed by Western blotting for P-MLKL, MLKL, P-RIPK3, RIPK3, 3D-Flag, and β-actin. **(C)** The analysis of the gray value ratio of P-RIPK3/β-actin in PK-15 cells 24 h after transfection with 3D-Flag plasmid. **(D)** The analysis of the gray value ratio of P-MLKL/β-actin in PK-15 cells 24 h after transfection with 3D-Flag plasmid. Data were presented as the mean ± standard deviation from three independent experiments. Statistical significance was denoted as follows: ***, *p* < 0.001.

### 3.6 FMDV 3D protein interacts with RIPK3

FMDV 3D protein activates the RIPK3/MLKL signaling pathway. To further investigate the relationship between the FMDV 3D protein and RIPK3/MLKL, co-immunoprecipitation (Co-IP) assays were first performed. The results showed that the 3D protein interacted with RIPK3 (Figures 6A,B), whereas no interaction was detected between the 3D protein and MLKL (Figures 6C,D). Subsequently, indirect immunofluorescence assays were conducted to examine the subcellular localization of 3D and RIPK3. The results revealed co-localization of the 3D protein with RIPK3 (Figure 6E). These findings suggest that the FMDV 3D protein may activate the RIPK3/MLKL signaling pathway through its interaction with RIPK3. To explore the molecular basis underlying the interaction between FMDV 3D and RIPK3, we used AlphaFold3 to predict the complex structure of RIPK3 with FMDV 3D, with particular focus on the RHIM-containing region of RIPK3. As shown in the Figure 6F, the predicted complex suggests that FMDV 3D can directly associate with the RHIM motif of RIPK3, forming a defined interaction interface. A magnified view of the predicted interface further revealed that several residues of FMDV 3D are involved in contacting the RHIM region of RIPK3. Specifically, residues R320, H322, E324, and Y346 of 3D were predicted to localize at the interaction surface, while residues within the RHIM-containing segment of RIPK3, including N466, V469, and K471, were positioned toward the binding interface (Figure 6F).

**Figure 6:**
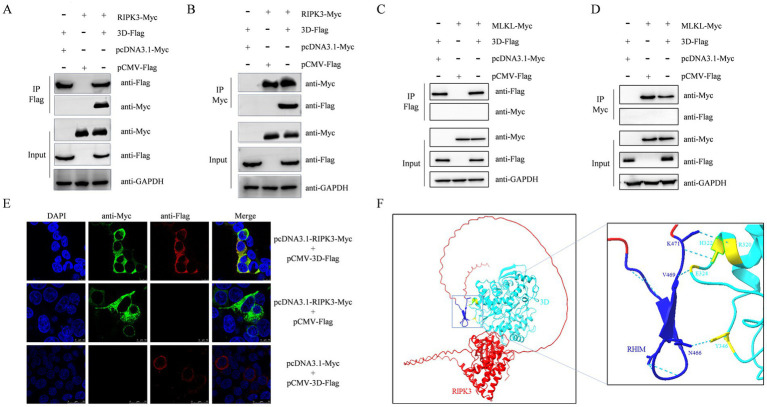
FMDV 3D protein interacts with RIPK3. **(A)** Co-IP assay in HEK-293 T cells. HEK-293 T cells were transfected with RIPK3-Myc and 3D-Flag or vector plasmids, and then cell lysates were immunoprecipitated with Flag antibody, followed by Western blotting with Myc and Flag antibodies. **(B)** Co-IP assay in HEK-293 T cells. HEK-293 T cells were transfected with RIPK3-Myc and 3D-Flag or vector plasmids, and then cell lysates were immunoprecipitated with Myc antibody, followed by Western blotting with Myc and Flag antibodies. **(C)** Co-IP assay in HEK-293 T cells. HEK-293 T cells were transfected with MLKL-Myc and 3D-Flag or vector plasmids, and then cell lysates were immunoprecipitated with Flag antibody, followed by Western blotting with Myc and Flag antibodies. **(D)** Co-IP assay in HEK-293 T cells. HEK-293 T cells were transfected with MLKL-Myc and 3D-Flag or vector plasmids, and then cell lysates were immunoprecipitated with Myc antibody, followed by Western blotting with Myc and Flag antibodies. **(E)** Co-localization of 3D and RIPK3. HEK-293 T cells were cultured on the bottom of glass cell culture dish for 12 h, and then transfected with 3D-Flag or RIPK3-Myc plasmids for 24 h. Transfected cells were analyzed by immunofluorescence staining with anti-Flag (red), anti-Myc (green) and DAPI (blue) under confocal microscopy. **(F)** Structural prediction of the interaction between FMDV 3D and the RHIM-containing region of RIPK3 by AlphaFold3. Left, overall model of the RIPK3-3D complex, showing FMDV 3D in close proximity to the RHIM-containing region of RIPK3. Right, enlarged view of the predicted interaction interface. Candidate interface residues include R320, H322, E324, and Y346 in 3D, and N466, V469, and K471 in RIPK3. Dashed lines indicate predicted inter-residue contacts.

## 4 Discussion

Normal or stimulated tissues and cells frequently undergo cell death. This process is essential for maintaining tissue and cellular function and morphology. To date, the major known modes of cell death include apoptosis, autophagy, necroptosis, anoikis, ferroptosis, phagocytosis, immune killing, necrosis, mitotic catastrophe, entosis, excitotoxicity, pyroptosis, oncosis, paraptosis, aborted apoptosis, and cuproptosis (28–32).

Viral infection often leads to cell death, and apoptosis is the most extensively studied type of cell death. Studies have shown that infection with reovirus (RV) (33, 34), SARS-CoV-2 (35–37), influenza A virus (IAV) (38–40), herpes simplex virus 1 (HSV-1) (41), herpes simplex virus 1 (HSV-2) (42), human cytomegalovirus (HCMV) (43), and vaccinia virus (VACV) (44) can induce or inhibit necroptosis.

Research on the modes of cell death induced by FMDV has focused on apoptosis and autophagy, whereas necroptosis has not been reported to date. It has been shown that FMDV VP1, 2C, and 3C proteins play important roles in inducing apoptosis and pyroptosis (45–48). Previous work from our laboratory found that the FMDV capsid protein VP2 induces autophagy and promotes viral replication by interacting with HSPB1 and activating the EIF2S1-ATF4 pathway (49); it was also found that FMDV infection utilizes RIG-I to transmit signals to STING, specifically activating PERK, which in turn mediates ISR to activate endoplasmic reticulum autophagy, and that ER autophagy negatively feedback-regulates STING degradation (50). The results of the present study demonstrate that FMDV infection induces necrosis in PK-15 and BHK-21 cells. Further analysis revealed that FMDV infection induces phosphorylation of RIPK3 and MLKL, thereby activating the RIPK3/MLKL signaling pathway and leading to necroptosis. This phenomenon occurred under natural infection conditions without inhibition of caspase activity. In contrast, previous studies have found that some viruses induce necroptosis only after caspase activity is inhibited, suggesting that multiple forms of cell death may be triggered during FMDV infection. However, the precise mechanism by which FMDV induces necroptosis in PK-15 and BHK-21 cells requires further in-depth investigation.

Functional experiments demonstrated that inhibition of RIPK3 phosphorylation by GSK’872 or inhibition of MLKL phosphorylation by NSA significantly reduced FMDV VP1 protein expression and viral titers, suggesting that the necroptosis pathway plays a promoting rather than restrictive role in FMDV replication. This finding is consistent with the dual role of necroptosis in viral infections—in some cases, necroptosis restricts virus spread by eliminating infected cells (e.g., HSV-1, RVA) (13, 51), while in others it is exploited by viruses to promote their own replication (e.g., CA6) (52). FMDV may induce necroptosis by activating the RIPK3/MLKL pathway, utilizing membrane permeabilization to release intracellular contents and create a microenvironment favorable for viral release and spread. Furthermore, the proinflammatory properties of necroptosis may contribute to FMDV-induced pathological inflammation, consistent with the tissue damage and inflammatory responses observed during FMDV infection.

The FMDV 3D protein, a virus-encoded RNA-dependent RNA polymerase, serves as the catalytic component for the synthesis of both positive and negative-sense viral RNA genomes and plays an essential role in the viral life cycle. In picornaviruses, the 3D protein of coxsackievirus A6 (CA6) has been shown to interact with RIPK3 (52). Infection with group A rotavirus (RVA) induces necroptosis, and transfection of the virus-encoded non-structural protein NSP4 activates RIPK1, RIPK3, and MLKL (51). To further investigate whether FMDV-encoded proteins can activate the RIPK3/MLKL signaling pathway, PK-15 cells were transfected with constructed eukaryotic expression plasmids encoding FMDV VP0, VP1, VP2, VP3, 2B, 2C, 3A, 3C, 3D, and L, respectively. It was found that the 3D protein significantly upregulated the phosphorylation levels of RIPK3 and MLKL, thereby activating the RIPK3/MLKL pathway. Moreover, 3D was found to interact with RIPK3, which may be critical for activation of the RIPK3/MLKL pathway.

The finding that FMDV 3D interacts with RIPK3 but not directly with MLKL is consistent with the established hierarchy of necroptosis signaling. RIPK3 functions as the central kinase that phosphorylates MLKL upon activation, whereas MLKL is the terminal effector that executes membrane permeabilization. From a structural perspective, the RIPK3 interaction interface typically involves RHIM domains, which are characterized by conserved β-strand forming tetrapeptide motifs. Sequence analysis of FMDV 3D revealed no canonical RHIM motif, suggesting that 3D may interact with RIPK3 through a novel interface or via an adaptor protein. Notably, AlphaFold3 structural prediction revealed that the 3D protein can directly associate with the RHIM-containing region of RIPK3, with residues R320, H322, E324, and Y346 of 3D contacting residues N466, V469, and K471 of RIPK3 at the interface. While these predictions provide valuable structural clues for further experimental validation, the exact binding mode between 3D and RIPK3, as well as the mechanism by which 3D activates RIPK3 kinase activity, awaits elucidation by structural biology approaches including co-crystallization or cryo-EM analysis.

This study uncovers a previously unrecognized function of the FMDV 3D protein as an inducer of necroptosis, extending beyond its classical role as an RNA polymerase and deepening our understanding of its involvement in virus–host interactions. In recent years, the non-polymerase functions of picornavirus 3D proteins have drawn increasing attention (6), and the present findings add a new dimension to this emerging field. Nevertheless, several limitations should be acknowledged. First, the precise molecular mechanism by which the 3D protein activates RIPK3—for instance, whether it involves RIPK3 oligomerization or amyloid fibril formation—remains to be elucidated. Second, the physiological relevance of FMDV-induced necroptosis and its contribution to infection *in vivo* require validation using animal models.

In summary, this study demonstrates that FMDV infection induces necroptosis in PK-15 and BHK-21 cells through the interaction of the 3D protein with RIPK3, which activates the RIPK3/MLKL signaling pathway, and that this pathway promotes FMDV replication (Figure 7). These findings not only broaden the landscape of host cell death modes triggered by FMDV but also offer new insights into the role of necroptosis in viral infections and provide a theoretical basis for the development of anti-FMDV therapeutics. Future research directions may include screening small-molecule inhibitors targeting the 3D–RIPK3 interaction interface, investigating the pathological significance of FMDV-induced necroptosis in animal models, and exploring the crosstalk between this pathway and other cell death modalities.

**Figure 7:**
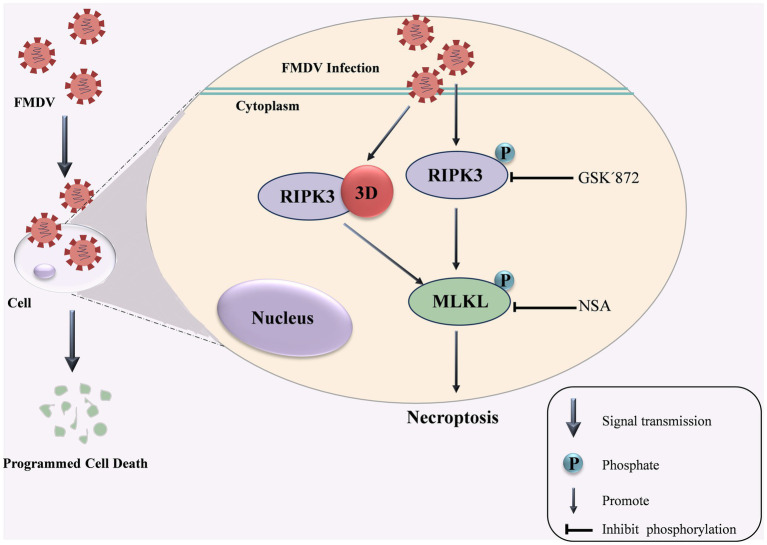
Proposed model: FMDV 3D interacts with RIPK3 to induce necroptosis. Schematic representation shows the model of FMDV non-structural protein 3D interacts with RIPK3 to induce necroptosis in PK-15 and BHK-21 cells.

## Data Availability

The original contributions presented in the study are included in the article/supplementary material, further inquiries can be directed to the corresponding author.
